# Snake Fungal Disease (Ophidiomycosis) in Northern Pine Snakes (*Pituophis melanoleucus melanoleucus*) in New Jersey: Variations by Year, Sex, and Morphological Sampling Site

**DOI:** 10.3390/jof11030206

**Published:** 2025-03-06

**Authors:** Joanna Burger, Christian Jeitner, Robert T. Zappalorti, John Bunnell, Kelly Ng, Emile DeVito, David Schneider, Michael Gochfeld

**Affiliations:** 1 Cell Biology and Neuroscience, Ecology, Evolution and Natural Resources, Rutgers University, 604 Allison Road, Piscataway, NJ 08854, USA; kelng@dls.rutgers.edu; 2 Center for Environmental Exposures and Disease, and Environmental and Occupational Health Sciences Institute, Rutgers University, Piscataway, NJ 08854, USA; mg930@eohsi.rutgers.edu; 3 New Jersey Pinelands Commission, New Lisbon, Pemberton, NJ 08068, USA; christian.jeitner@pinelands.nj.gov (C.J.); john.bunnell@pinelands.nj.gov (J.B.); 4 Herpetological Associates, Inc., Pemberton, NJ 08068, USA; rzappalort@aol.com (R.T.Z.); dschneider@herpetologicalassociates.com (D.S.); 5 Conservation Foundation of New Jersey, Far Hills, NJ 07931, USA; emile@njconservation.org; 6 Rutgers Biomedical and Health Sciences, Rutgers University, Piscataway, NJ 08854, USA

**Keywords:** snake fungal disease, SFD, *Ophidiomyces ophidiicola*, ophidiomycosis, yearly differences, hibernation lesions, skin lesions, pine snake

## Abstract

*Ophidiomyces ophidiicola*, the fungus causing Snake Fungal Disease (SFD) or ophidiomycosis, is prevalent in North American snakes and can have deleterious population effects. Northern pine snakes (*Pituophis melanoleucus melanoleucus*) in New Jersey often test positive for ophidiomycosis. In this paper, we use qPCR to examine changes in prevalence from 2018 to 2023, and differences by age, sex, and morphological sampling locations. We swabbed ventral surfaces, head, and cloaca of snakes, and lesions and eyes if there were clinical ophidiomycosis signs. A snake was considered positive if any site was positive by qPCR. The prevalence was 47% (2018), increased to 100% (2022), but declined to 46% in 2023. The prevalence was highest in snakes with lesions (46–100%); head swabs had the lowest rates. The more lesions a snake had, the more likely it was that at least one would be positive. Males had significantly more lesions than females, but the prevalence was similar. In 2023, the prevalence of *O. ophidiicola* was low, but the prevalence of lesions did not decrease as markedly. We discuss the temporal changes in the positivity for *O. ophidiicola* and its implications for ophidiomycosis effects, suggesting that the fungus is endemic in this population.

## 1. Introduction

Emerging infectious diseases have the potential to impact all nodes on the food chain, including top-trophic level predators, including humans. Some species and populations have been harmed and suffered global population declines. The most often-cited examples of emerging infectious diseases that have drastically decreased populations are Chytridiomycosis in amphibians (caused by *Batrachochytrium dendrobatidis* and *B. salamandrivorans*) and white-nose syndrome (caused by *Pseudogymnoascus destructans* [1,2,3]). In snakes, snake fungal disease (SFD) or ophidiomycosis, caused by *Ophidiomyces ophidiicola*, has emerged as potentially harmful, causing death in some individuals [4,5,6,7]. Ophidiomycosis has been identified in several snake taxa not only in the U.S., but in Europe, Asia, and Australia [8,9,10]. In some species of snakes, it can cause severe disease and mortality [11]. Scientists and conservationists are concerned that ophidiomycosis may cause local extinctions of isolated populations, particularly given fragmentation of habitats [11,12]. In the literature, reports of ophidiomycosis began appearing around 2013 [6]; the first confirmed case of ophidiomycosis in wild snakes in America was in the Eastern Massasauga rattlesnakes (*Sistrurus catenatus*) in Illinois in 2008 [7,13] and later in Michigan [4]. Ophidiomycosis has since been confirmed in many other wild North American snakes [14,15,16,17], and there are reported cases of *O. ophidiicola* as early as 1945 from museum specimens [18]. There continue to be reports of ophidiomycosis in a number of snakes species, although it is most common in Colubridae and Viperidae and in semiaquatic species of terrestrial snakes [19,20].

Snakes in the wild can exhibit skin lesions, behavioral alternations, abnormalities of reproductive physiology, thermoregulation, and other abnormalities that are associated with ophidiomycosis [6,21,22,23,24]. Snakes infected in the laboratory with *O. ophidiicola* develop the same kind of skin lesions and abnormalities as do snakes in the wild [21,22,23]. Laboratory experiments demonstrate that *O. ophidiicola* can grow at the low temperatures that snakes are exposed to in hibernacula during the winter, suggesting that hibernacula may be a source of transmission and increased infection [19]. There are more reports of ophidiomycosis in the winter and following hibernation than at other times of the year [24,25,26]. The presence of *O. ophidiicola* was more frequent after hibernation, and declined thereafter [27], although seasonal variations may not always be directional [28]. Early seasonal basking has also occurred with snakes infected with *O. ophidiicola* [29]. Hibernacula may serve as winter reservoirs for *O. ophidiicola* when snakes congregate in large groups. Transmission could occur from snake to snake (either the same or a different species), or from soil to snake [30,31]. Campbell et al. [32] found that *O. ophidiicola* occurred in the soil of Northern pine snakes (*Pituophis melanoleucus melanoleucus*) hibernacula in New Jersey, both in the main chambers and beneath snakes inside chambers. We subsequently reported a higher positivity rate of snakes that hibernated on soil that tested positive for ophidiomycosis than on soil that did not test positive [33]. For example, over 85% of the pine snakes that tested positive for ophidiomycosis were hibernating on soil in chambers where soil also tested positive for *O. ophidiicola* [33]. We found that the prevalence of ophidiomycosis in pine snakes from New Jersey increased from 58% in 2018 to 83% in 2020 and 70% in 2021, suggesting cause for concern for this population of pine snakes, but also for other species traditionally using the same hibernaculum year after year. Timber rattlesnakes (*Crotalus horridus*), corn snakes (*Pantherophis guttatus),* and black racers (*Coluber constrictor*) also use our hibernacula and tested positive for *O. ophidiicola* (JB, unpublished data). We were concerned that ophidiomycosis might be causing morbidity or mortality among pine snakes and other species, which may potentially cause population declines.

In this paper we examined the prevalence of *O. ophidiicola* in free-ranging Northern pine snakes in the New Jerey Pinelands. We sampled hibernating snakes in late February or early March, about a month or more before they normally emerge. Our objectives were to determine: (1) temporal trends in the overall prevalence of ophidiomycosis, (2) if the overall prevalence was similar among morphological tissue sampling locations on the snakes, (3) if number of snakes with lesions and prevalence of lesions were related, and (4) if there were sex and age differences in prevalence of ophidiomycosis. Positivity was determined by qPCR, and any lesion or scale discoloration was swabbed. There is considerable variation in the literature about the best methods of successfully and economically determining ophidiomycosis by clinical means, by laboratory experiments, or by qPCR. Thus, after our pilot study (2018), we sampled several different sites per snake, including ventral surface, head, cloaca, and all lesions (2019–2023). Lesions, which we had previously recorded as “hibernation sores” were swabbed separately. A sample was designated as positive if any swab tested positive by qPCR (e.g. head, cloaca, ventrum, lesions). 

Pine snakes in New Jersey may be particularly vulnerable to ophidiomycosis because they usually hibernate together in hibernacula that are 1–3 m below ground for five or more months in the winter [34]. We began studying the ecology and behavior of the Northern pine snakes in the late 1970s because little was known about the species, and NJ was concerned about protecting their populations. The Pine Barrens was undergoing extensive human development, although it had been declared a Pinelands National Reserve by Congress [35]. We often followed or radio-tracked pine snakes to holes, and assumed they were hibernating there. We began excavating hibernacula in March 1985 [34,36]. We found up to 30 pine snakes in one hibernaculum, although the number is usually lower [37]. Pine snakes emerge from hibernation in late March-April, mate in April-May, and nest in late June-early July.

All snakes are PIT-tagged, measured, and weighed. In 2018, we conducted a pilot study on ophidiomycosis, and since 2019, we have swabbed all snakes encountered in our dens. Since the 1980s, we have noted a few damaged, discolored, or ragged scales on pine snakes, which we identified as “hibernation lesions”. Since snakes reappeared the next year in hibernation, with or without lesions, and we found no dead snakes in our hibernacula, we assumed the damaged scales were simply due to abrasions acquired during digging or movement in their chambers and were indeed lesions resulting from hibernation [34,38]. Since pine snakes shed several times a year, and snakes we found in the summer seldom had “hibernation lesions,” we were unconcerned. The reports of ophidiomycosis in the literature alerted us to begin swabbing and testing for *O. ophidiicola* in 2018. We had not found snakes with lesions or deformities of head, eyes, or mouth as depicted in other studies [11,17], so we were not anticipating finding ophidiomycosis.

## 2. Materials and Methods

### 2.1. Study Species and Study Sites

**Figure 1:**
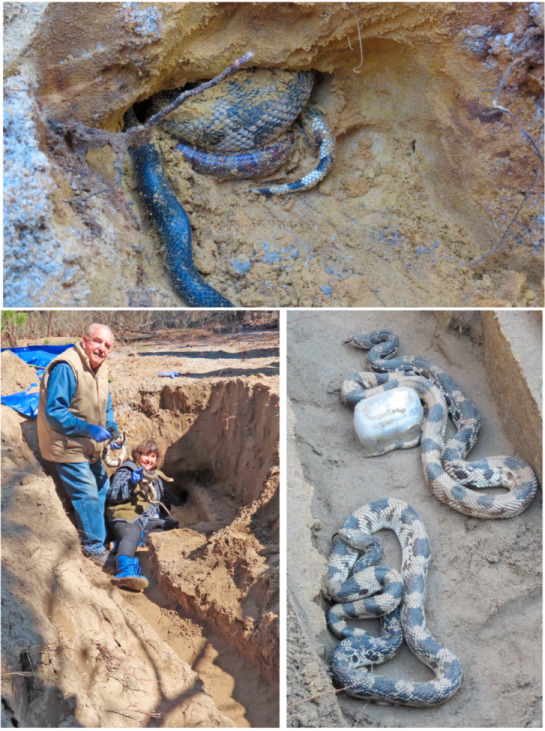
Pine snakes dig hibernacula that are 3 m or more long. The photo on the **bottom left** shows the main chamber and tunnel. We each hold a snake just pulled out of the lower area. In the **bottom right** photo are two snakes in the main chamber, with a plastic box holding a temperature recorder. In the **top** photo, a black racer, corn snake, and pine snake are in a joint chamber at the end of a tunnel.

We designated a pine snake that hatched in late August or early September as age 0 when encountered in the fall of its hatching year [36]. If we encountered the same snake in a hibernaculum when they were excavated in early March (Figure 1), it was designated as one-year-old. In the following March, for example, this snake (ca 18 months old) was designated a two-year-old.

We conducted a pilot study of ophidiomycosis with ventral swabs of 12 individual pine snakes in 2018 using careful field hygiene procedures to avoid introducing or spreading the fungus if it was present. Based on finding 58% positive qPCR detections, we began a more detailed study in 2019 of different morphological sampling sites (ventral, head, cloaca, lesions [26]). Since then, we excavated three “hibernacula complexes” of three to five dens each in Burlington and Ocean County, New Jersey. We do not divulge the exact locations of these dens due to high levels of poaching [34,38]. Not all dens are occupied each year (although the complexes are). Currently, each active den has 1 to 15 pine snakes, depending upon the sampling year. Our overall approach after 2018 was to examine the prevalence of *O. ophiodiicola* detections in all free-ranging snakes located in our excavated hibernacula from 2019 to 2023. We collected swab samples from snakes as they were removed from the hibernacula. All personnel changed gloves between every snake, and all equipment was sanitized between dens complexes. Snakes were returned to their dens the same day.

Our field and laboratory studies were approved by the Rutgers University Animal Care and Use Committee (permit # E6-017, renewed every three years), by permits from the New Jersey Department of Environmental Protection (Endangered and Nongame Species Program, renewed every year) and New Jersey Parks and Forestry, and with permission from private landowners. The snake’s welfare is always our greatest concern. No pine snake was injured during any of our snake digs.

### 2.2. Sample Collection

Our overall protocol was to remove snakes from each hibernacula chamber, swab them, and return them to their hibernaculum after all snakes were processed and the hibernacula was rebuilt. Initially, we excavated natural hibernacula, but thereafter we rebuilt the hibernacula [34], allowing the snakes to leave the main constructed chamber and dig their own side chambers into the virgin sand. Subsequently, we carefully dug down until we reached the roof of the constructed hibernaculum, removed any snakes, and then followed their tunnels until we reached any snakes that were in chambers dug out from the main chamber (Figure 1). Snakes were carefully removed and passed to the ophidiomycosis sampling station. All personnel changed gloves between each snake. Snakes were examined for lesions, bumps, discolored scales, and other abnormalities, and swabbed immediately. The snakes were then measured, weighed, and a PIT tag was inserted if they were new. All personnel involved in removing snakes from their hibernation chambers, transporting them to the swabbing station, swabbing them, processing them, or otherwise handling the snakes changed nitrile gloves between each snake. In the 2018 pilot study of 12 pine snakes, only the entire ventral surface from the head past the cloaca to the tail tip was swabbed in a single pass. In 2019–2023, we collected a ventral, head, and cloaca swab, and a separate swab for each lesion for all snakes. For each snake, we swabbed each different part of the body with sterile polyester-tipped swabs that were premoistened with sterile deionized water. We sampled the ventral surface using a swab from the head to the tip of the tail, excluding any lesions present [26]. Additional swabs were taken of the head and from each individual lesion. Lesions included any discolored or ragged margins of scales, abraded or crusted scales, or swollen or mounded scales. Swabs were stored in screwcap tubes, placed on ice in the field, and stored frozen at −30 °C in a freezer until analysis.

**Figure 2:**
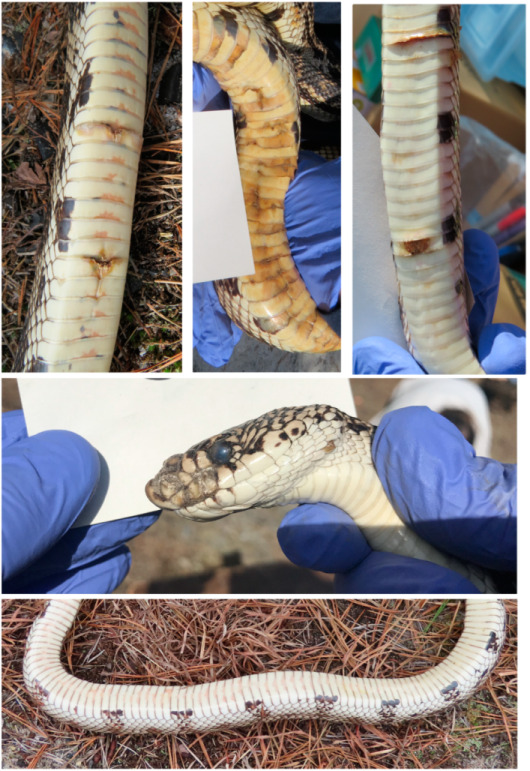
Samples of lesions that tested positive for ophidiomycosis in pine snakes in New Jersey (**top** three), a sore on the head and cloudy eye (**middle**, tested positive), and a pristine ventral surface of a pine snake without lesions (**bottom**).

We note that we were sampling external bumps, lesions, and cloudy eyes, which are signs of *ophidiomycosis* (Figure 2) [6,39]. We did not examine internal effects by microscopy, which may have found arthrospores or mycelium. Lesions around the head and mouth can lead to internal infection and death [4,5,6,7].

### 2.3. Determining Ophidiomycosis by Quantitative Polymerase Chain Reaction (qPCR)

The same methodology for the determination of ophidiomycosis was used in all years, and ophidiomycosis was determined by qPCR in the same laboratory [26,32]. The presence of *O. ophiodiicola* was determined for 146 Northern pine snakes from the NJ Pinelands by extraction of nucleic acid from swab samples using a specific qPCR targeting the internal transcribed spacer region of the fungus, as described by Bohuski et al. [40]. Samples were defined as positive for *O. ophiodiicola* if they had 15 or more copies of target DNA (based on standard curves on each PCR run). A snake was considered positive in this study if it was PCR positive for any one of the swab samples (e.g., ventral, head, lesion, cloaca). In this study, we assume that with PCR there are very few false positives, which may arise from sample contamination. False negatives can arise from sampling techniques in the field or sample handling before or after reaching the laboratory. Some of the variability noted may be due to such test errors. Further information on the methodology can be found in Burger et al. [26,33].

### 2.4. Statistical Analysis

We used non-parametric tests (Kruskal-Wallis X^2^ test or Fisher Exact Test, PROC NPAR1WAY) to determine differences among swabbing locations and lesions [41,42,43]. These non-parametric tests were used because they are more conservative and are best suited for small datasets with binary outcomes [43,44]. A chi-square test of homogeneity was used to evaluate the relationship between years. The mean scores for qPCR-positive and qPCR-negative lesions were compared by Kruskal–Wallis. *p* values of 0.05 and below were considered significant.

## 3. Results

### 3.1. Yearly Variations

**Figure 3:**
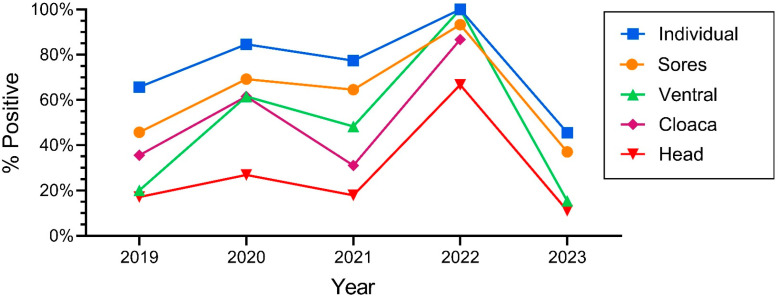
Percentage of snakes that were positive for *O. ophiodiicola* for the different morphological (body) sampling sites on Northern pine snakes from the New Jersey Pinelands (2019–2023).

One of the main objectives of this study was to determine if there were yearly variations, and whether there continued to be increases over time in the prevalence of ophidiomycosis. The number of snakes varied over the years; declines in numbers occurred in 2022 but rebounded in 2023 (Table 1). The percentage of snakes that tested positive with qPCR also varied significantly by year: ophidiomycosis increased markedly from 2018 to 100% in 2022 and decreased by half in 2023 (Table 1). Similarly, the positivity rate for individual sampling sites varied by year, with the rates being lower in 2023 compared to the earlier years. The prevalence for snakes overall (e.g., at least one swab on a given individual was positive) generally tracked the rates of positivity for individual tissues (ventral swabs, head, cloaca, lesions) (Figure 3). The percentage of snakes with a positive ophidiomycosis lesion also varied by year (Table 1).

Since all snakes have a ventral surface, a head, and a cloaca, we had swabs for all snakes; not all snakes had lesions, so there was a smaller sample for snakes with sores. Further, not all lesions were positive. The number of snakes with any lesions is shown in the middle section of Table 1. The percentage of snakes that had at least one lesion that was positive for ophidiomycosis varied significantly by year (and ranged from 37% to 93%). Thus in 2022, nearly all snakes that had any lesion had at least one lesion that tested positive for ophidiomycosis. In contrast, in 2023, 14 snakes had lesions, and only 71% of those lesions tested positive (snakes often had more than one lesion) (Table 1).

### 3.2. Relationships Among Snakes and Lesions

**Figure 4:**
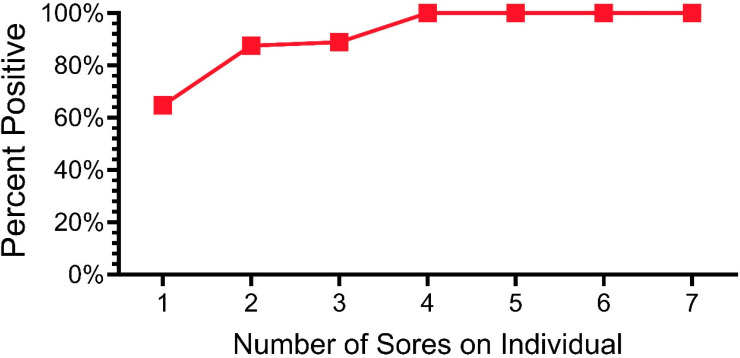
Percentage of pine snakes that were positive for *O. ophidiicola* by qPCR as a function of the number of lesions on individual snakes.

In the section above, we examined the percentage of snakes that tested positive from 2019 to 2023. Of the morphological sites examined (e.g., ventral, eye, cloaca, lesions), lesions had the highest percentage of qPCR positivity. One important question concerns the severity of lesions and whether any type or degree of damage or discoloration is indicative of ophidiomycosis. In a previous paper, we showed a lack of agreement among investigators about whether a lesion indicated ophidiomycosis and qPCR positivity [33]. We found that investigators could not accurately predict whether a lesion (or discoloration) would or would not test positive for *O. ophidiicola*. When we swabbed all lesions, not all were positive for *O. ophidiicola*. In this section, we examined lesions (all lesions combined, and lesions per snake). Figure 4 shows the relationship between the percentage of lesions that were positive and the number of lesions a snake had (2019–2023) for all snakes that had lesions. That is, over 63% of the snakes that had only one lesion had a positive lesion. In contrast, any snake with at least four lesions was likely to have at least one lesion test positive (Figure 4).

**Figure 5:**
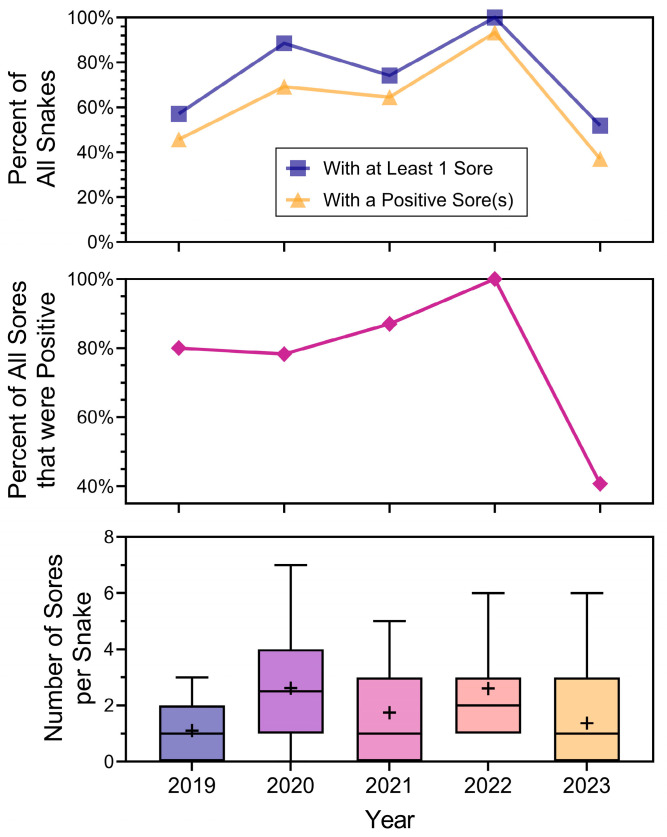
Summary of the relationship between the percentage of snakes with lesions (and those with at least one positive lesion) (**top**), overall positivity of all lesions by year (**middle**), and a box and whisker plot with the mean number of lesions per snake by year (**bottom**). The line inside the interquartile range (IQR) box indicates the median, + is the mean, and the whiskers extending from the IQR are the minimum and maximum values.

We then examined the relationship between snake lesions and the year of sampling (Figure 5). Figure 5 is arranged to present the main measurements by year: (1) percentage of snakes that had at least one lesion (top panel), and percent of snakes that had at least one positive lesion (obviously less than the percentage of lesions), (2) percentage of lesions that were positive by year (all lesions combined, middle panel), and (3) in the bottom panel, the number of lesions per snake with median, mean, variances, and ranges. It summarizes several different measures of lesions on snakes that are used in the literature. Separating whether lesions in general are positive, or whether at least one lesion on a snake is positive will begin to distinguish whether a lesion on a snake indicates ophidiomycosis. There were yearly variations in all these measures (Figure 5). The mean number of lesions per snake (that had lesions; bottom panel) indicates variability from year to year in the range and variances. Overall, however, there were still snakes with many lesions in 2023, but fewer were positive (middle panel of Figure 5).

### 3.3. Prevalence of Ophidiomycosis as a Function of Sex or Age

**Figure 6:**
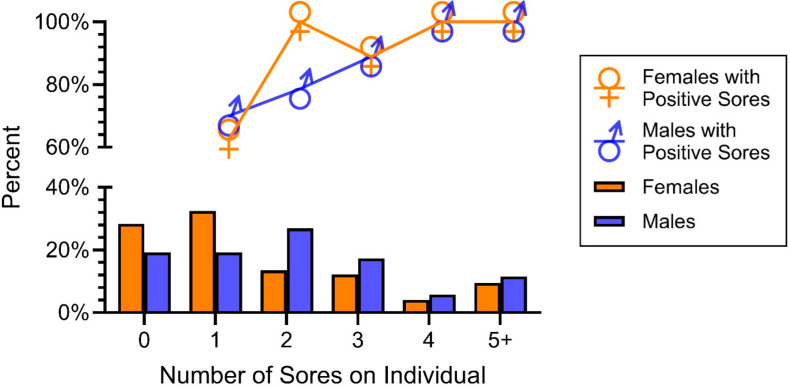
Prevalence of lesions in males and females (bottom graph) and percent with positive lesions. Males had significantly more lesions than females but not significantly more positive lesions.

Our final objective concerned determining if there were sex or age differences in the prevalence of *O. ophiodiicola*. When snakes were combined (2019–2023), there were no significant sex differences in the prevalence of *O. ophiodiicola* (Table 2). However, there were significant sex differences in how many lesions individual snakes had (Figure 6). On this graph, the percentages add up to 100% for females and 100% for males. Using a Chi-Square test, we compared whether there were sexual differences between two categories: no lesions or only one vs two or more lesions (Figure 6, X^2^ = 6,1, *p* > 0.01). Males had significantly more lesions than females overall; males had a mean of 2.6 lesions/snake and females had an average of 2.3 lesions/snake.

There were no clear age-related differences in the prevalence of *O. ophiodiicola*. Snakes of all ages tested positive, including a few individuals over 16 years of age (Table 3). Although Table 3 indicates the ages of breeding, it does not mean the snakes actually bred at those ages.

## 4. Discussion

### 4.1. Prevalence and Methodologies

Ophidiomycosis (SFD) is an emerging disease that requires not only well-defined sampling methods (tissues or number of swabs) and temporal patterns of sampling (once/year, seasonal), but clear definitions of what constitutes the disease. Both surveillance and pathogenicity are fundamental to conservation, population sustainability, and public health [5]. Clearly, there are clinical signs of ophidiomycosis, such as injured, damaged, or discolored snake scales, severe swelling of the head (or bumps), and invasion of underlying muscle and bone [27]. However, such clinical signs do not always test positive for *O. ophidiicola*; lesions do not always test positive for *O. ophidiicola*. For example, many papers report “clinical signs” of ophidiomycosis that did not test positive by qPCR [5,45], and we also found this in Northern pine snakes. In a previous study, we showed that the investigator’s perceptions of the clinical signs of ophidiomycosis do not significantly correlate with the qPCR detection of *O. ophidiicola* [33].

Previous data, and data presented in this paper on ophidiomycosis in Northern pine snakes suggest the importance of not assuming a snake is free from ophidiomycosis even if its skin looks relatively clean and unblemished, and conversely, some really discolored lesions do not test positive. Although the overall percentage of positives using qPCR detection was correlated for different tissues of pine snakes, this was not necessarily true for each snake. Not all obvious lesions, scale damage, or discoloration tested positive in our studies. Sometimes the most damaged scales did not test positive. Other authors also reported that lesions do not always test positive. For example, Haynes et al. [46] found that in free-ranging snakes in 34 species, 28% had skin lesions and 13% were positive. The conclusion from our studies to date is that swabbing should include several swabs of the head, ventral surface, cloaca, and all lesions, with subsequent qPCR detection of *O. ophidiicola*.

If any swab of a snake tested positive, we considered the snake positive. However, the degree of the disease in a particular pine snake was unclear. If only one of five lesions tests positive, and no other swabs test positive (e.g., head, cloaca, ventral), the question was: did the infected snake recover from ophidiomycosis, or was it heading toward an infection? These questions require careful laboratory examinations. The absence of snakes with obvious signs of severe illness (e.g., lesions on the head or around the mouth, or being vastly underweight) in our hibernacula may suggest a lack of disease even though they are infected with (or carrying) *O. ophiodiicola*.

### 4.2. Yearly Variations in Northern Pine Snakes

The most obvious source of variation in qPCR detection of *O. ophidiicola* in the 146 Northern pine snakes we tested was due to year and varied from 100% to 45.5% (in successive years). It is remarkable that the percentage that tested positive increased generally from 2018 to 2022 and declined dramatically in 2023. The decline was not due to the overall death of infected individuals since there were more snakes in our hibernacula in 2023 than in 2022 (and some of the same individuals were found in both years). In all our years of study, we have not found any dead snakes in our hibernacula whose deaths could be attributed to ophidiomycosis because all snake deaths in the hibernacula were hatchlings that died because they were crushed by larger snakes lying on top of them (hatchlings weigh about 50 g, while a large adult can weigh up to 1200 g [38]), or a few snakes died half-way down hibernation entrances where they froze. In contrast, in an experimental study with red corn snakes (*Pantherophis guttatus*), 87% of inoculated snakes died over the 70-day brumation experiment [23].

One hypothesis is that the prevalence of *O. ophidiicola* in pine snakes entering the hibernacula in the fall varies from year to year, for unknown reasons. More likely, we suggest that the reservoir of *O. ophidiicola* in the soil of pine snake hibernacula varies from year to year. Thus, the inoculation of snakes lying on that soil varies from year to year. Previous work from only one season indicated snakes that tested positive for *O. ophidiicola* were more likely to be found hibernating on soil that tested positive for *O. ophidiicola* than snakes that did not test positive. This begs the question, however, of why the reservoir varies from year to year. Either moisture or temperature differences may be the limiting factor; both may depend on the depth of the main chamber and the side chambers that snakes dig, as well as environmental factors. This requires considerably more experimentation for several years. An experiment with prairie rattlesnakes (*Crotalus viridis*) showed that *O. ophidiicola*-infected snakes exposed to lower temperatures had decreased survival [47].

Another unknown is how long *O. ophidiicola* remains viable in the soil of the hibernacula after the snakes emerge in the spring. After we dig up the hibernacula in the late winter, snakes are returned the same day to the reconstructed hibernacula, and they usually remain for a month or longer before they emerge in the spring. Snakes presumably move within the main hibernaculum chamber and may dig side chambers where infected snakes could inoculate the soil. We have some evidence that this may occur. In one year (2022) when three females laid clutches in nest chambers that arose from the main hibernacula chamber, we found that their eggshells tested positive for *O. ophidiicola*, as did some of the hatchlings themselves (eggs were subsequently hatched in the laboratory). Hatchlings that hatched from natural nests dug by the snakes in the nesting area (at some distance from hibernacula) did not test positive for *O. ophidiicola* in the fall, before they entered the hibernacula. These data suggest that the soil in the hibernacula remained inoculated with *O. ophidiicola* from early March (date of hibernacula dig) to at least early June–July (during egg-laying) (Burger, unpublished data). We note that no other hatchlings found in the fall that emerged from natural nests tested positive by qPCR [33]. The main conclusion, however, is that despite having all the snakes in hibernacula test positive for *O. ophidiicola* one year (2022), the following year only 45% tested positive. This sudden decrease in positivity may argue against *O. ophidiicola* remaining in the hibernacula from year to year. Harding et al. [9] used preserved *Nerodia* at university museums in Texas to show that the proportion of snakes showing signs of *O. ophidiicola* infection did not increase over space and time (a 29-month period). A more detailed long-term study of different species of snakes in eastern states might shed additional light on *O. ophidiicola* prevalence over time.

### 4.3. Sex Differences in Prevalence

When the five years were examined together (2019–2023), there were no sexual differences in the overall positivity rate, nor in whether they had at least one positive lesion. However, females had significantly fewer lesions than males. Our previous work indicated that males had a higher *O. ophidiicola* prevalence [26]. Similarly, Lind et al. [24] noted that pregnant females were not more likely to test positive for *O. ophidiicola* than other snakes but were less likely to exhibit clinical signs of the disease compared to males. We have no obvious reason why males would have more lesions than females but not a higher positivity rate of at least one lesion. It is possible that females have fewer lesions than males because, during the latter stages of egg development, they spend time basking in the sun rather than moving underground through mammal tunnels or simply resting underground. The relationship of positivity of *O. ophidiicola* to sex and age requires considerably more investigation with marked snakes followed seasonally; positivity for *O. ophidiicola* has generally been higher following hibernation and decreases seasonally [20], particularly after snakes shed infected skin.

### 4.4. Emerging Infection or Endemic in Pine Snake Populations

The meaning of “emerging infectious disease” is unclear, and to us, it can mean many things: (1) a truly new, previously undiscovered disease; (2) a previously undiscovered disease that has only recently been discovered; (3) an endemic disease that went undiscovered until recently, and may have been present for decades, or some combination of these. Rachowicz et al. [48] recently defined it for wildlife as fitting within these categories but includes recently evolved organisms. The literature on such diseases in human exposures, as defined by the U.S. Environmental Protection Agency (EPA [49]), refers to “chemicals of emerging concern” just because of the nature of these differences.

The examination of captive snakes [50] and museum specimens [18] have documented the presence of *O. ophidiicola* from the 1940s. We found that the prevalence of *O. ophidiicola* by qPCR testing in NJ pine snakes ranged from 45.5% to 100%. Notably, Sperry et al. [51] reported that three of seven individual Louisiana pine snakes (*Pituophis ruthveni*) tested positive for *O. ophidiicola*. In a study in central New Jersey, Mark et al. [52] reported a rate of 26% for Eastern copperhead (*Agkistrodon contortrix*). Several authors have conducted broad-ranging studies of prevalence rates in free-ranging snakes. Dillion et al. [27] reported rates as high as 28% in Eastern fox snakes (*Pantherophis vulpinus*), with prevalence in lesions being 40%. Allender et al. [5] tested 657 individual snakes of 58 species in 31 U.S. states and identified a prevalence of 17% *O. ophidiicola* DNA. While these reports represent a large sample size, the sample for individual species is low, and variability of individual species cannot be inferred, nor can temporal patterns. There have been some high rates for some individual species, such as water snakes (*Nerodia sipedon* (73%)) and Eastern rat snakes (*Pantherophis alleghaniensis*, 70%, [53]), although the highest rate reported was 94% for adult *Nerodia erythrogaster transversa* [45,54,55]. Davy et al. [45] “diagnosed or suspected” ophidiomycosis in 441 of 2384 snakes from Canada, with mortality in about 10% of those with the disease, and concluded the disease is a previously unrecognized endemic organism rather than a novel pathogen. Similarly, Allender et al. [5] and Haynes et al. [46] concluded that ophidiomycosis is endemic, at least in the Eastern United States. We also concluded it was endemic in Northern pine snakes living in the New Jersey Pinelands [26,33].

For us, the question is not whether it is endemic, because “hibernation lesions” were present in hibernating Northern pine snakes in New Jersey when we began our study in the late 1970s. While these lesions were not tested for *O. ophidiicola*, the rather high prevalence of current lesions for *O. ophidiicola* suggests it has been present. Our field notes from individual snakes prior to 2018 noted up to three snakes in some years that had bad “hibernation sores”, but in most years no lesions were notable enough to record. That is, there were sometimes slight scale discolorations, but not abrasions, ragged or broken, or darkened scales. Since we found the same snakes in subsequent years, we were not overly concerned. Our impression upon digging up pine snakes in hibernacula for 40 years is that the percentage of snakes with lesions has increased, and the number and severity of sores per snake have increased in the last decade. We base our impression on the relative lack of field notes concerning lesions, although we have noted lesions as far back as 1987.

The data from the present study clearly show great yearly variation in the percentage of snakes that test positive for *O. ophidiicola* using qPCR detection. Furthermore, there is a very high rate of philopatry to hibernacula complexes in our study, and particular hibernacula have been used continuously for decades by snakes [34,36]. It thus seems likely that *O. ophidiicola* is endemic in this population. The question now is whether the apparent increase in prevalence (and severity) observed in the last few years is real, why this is happening now, and whether it will decline to a previous level. The possible causes for these shifts (e.g., temperature, moisture) are under investigation, but these require long-term studies and remote monitoring of conditions in the hibernacula.

## 5. Conclusions

The presence of *O. ophidiicola* in captive snakes on several continents, followed by its detection in wild snakes, suggests that the pet trade may be an important component of the worldwide spread because of the unsanitary handling and trade of snakes [56]. *O. ophiodiicola* has been present in wild snakes for decades, with a high prevalence of *O. ophidiicola* in the winter in hibernation. Although at present it appears to be endemic in the pine snakes we studied, as well as in many different species studied by several other authors [5,26,45,55], it does not mean it does not have the potential to become a major cause of declines, locally or regionally, in some species. We note that the prevalence in our pine snake population has varied significantly over a 6-year period, and not always in the same direction. The rapid decline in *O. ophidiicola* positivity from 2022 to 2023 in pine snakes, without a similar decline in the number of clinical signs (lesions), suggests the recovery of individual snakes. The variability in this population also suggests the need for consistent long-term monitoring of *O. ophidiicola* in some species, along with morbidity or mortality information to provide early warning of any significant change in prevalence or severity.

## Figures and Tables

**Table 1:** Percentage of Northern pine snakes (aged 1 year and older) testing positive for *O. ophiodiicola* in 2019 to 2023 (n = 134). A chi-square test of homogeneity was used to evaluate the relationship between years (n = 134). In 2018, 58% of 12 snakes tested were positive by qPCR ventral swabs (including lesions [26]). NS = not significant.

	2019	2020	2021	2022	2023	X^2^
Number of Snakes	35	26	31	15	27	
% Positive Individuals	65.7%	84.6%	77.4%	100.0%	45.5%	21.5 (0.0002)
% Positive Ventral	20.0%	61.5%	48.3%	100.0%	15.4%	39.6 (0.0001)
% Positive Head	17.1%	26.9%	17.9%	66.7%	11.1%	19.2 (0.0007)
% Positive Cloaca	35.5%	61.5%	31.0%	86.7%	- ^a^	16.2 (0.001)
% Positive Lesions	45.7%	69.2%	64.5%	93.3%	37.0%	3.0 (NS)
Number of Snakes with Lesions	20	23	23	15	14	
% of Snakes with Lesions	57.1%	88.5%	74.2%	100%	51.9%	
% of These Snakes with at Least one Positive Lesion	80.0%	78.3%	87.0%	93.3%	71.4%	23.4 (0.0001)
Number of All Lesions (2019–2023)	34 ^b^	68	54	39	37	
% Positive Lesions	73.5%	82.4%	79.6%	95.0%	59.4%	15.2 (0.004)
Mean Number of Lesions/Snake ^b^	1.1 ± 1	2.6 ± 1.9	1.7 ± 1.7	2.6 ± 1.7	1.4 ± 1.7	

^a^ No cloacal samples collected in 2023. ^b^ Of the snakes with at least one lesion.

**Table 2:** Percentage of pine snakes aged 1 and older testing positive for *O. ophiodiicola* as a function of sex (n = 131 snakes). Note: 3 snakes did not have a sex associated with them. NS = not significant.

	Male		Female		
	N	% Positive	N	% Positive	X^2^
Any Positive	54	79.6%	77	67.5%	2.3 (NS)
Ventral	54	51.9%	74	37.8%	2.5 (NS)
Cloaca	43	58.1%	58	41.4%	2.8 (NS)
Head	54	18.5%	74	28.4%	1.7 (NS)
Individuals with Lesions	42	83.3%	53	81.1%	12.2 (NS)
Total Lesions	110	75.5%	121	82.6%	1.8 (NS)

**Table 3:** Percentage of Northern pine snakes that tested positive by age (in years), based on 143 snakes tested from 2018 to 2023 in the New Jersey Pinelands.

Age Group	N (Number of Snakes)	Number of Snakes Positive	% Positive Per Age Group	Life Stage
1 to 3	50	32	64.0	Hatchling and Juveniles
4 to 6	18	17	94.4	Initial Breeding
7 to 9	18	12	66.7	Breeding
10 to 12	23	15	65.2	Breeding
13 to 15	20	13	65.0	Breeding
16 to 18	10	8	80.0	Breeding
19 to 25	4	3	75.0	Breeding ^a^
**Total**	143	100		

^a^ Unclear if pine snakes breed at this age as two in the laboratory did not lay fertile eggs at this age.

## Data Availability

Data are available from the senior author upon reasonable request.
